# PROVIDING INPATIENT CARE BEYOND HOSPITAL WALLS: GEOGRAPHIC FACTORS IN ACUTE HOSPITAL CARE AT HOME WAIVER PROGRAMS

**DOI:** 10.1093/geroni/igac059.084

**Published:** 2022-12-20

**Authors:** Emily Franzosa, Ksenia Gorbenko, Abigail Baim-Lance, Gabrielle Schiller, Heather Wurtz, Sybil Masse, Katherine Ornstein, Bruce Leff

**Affiliations:** Icahn School of Medicine at Mount Sinai, New York City, New York, United States; Icahn School of Medicine at Mount Sinai, New York City, New York, United States; Icahn School of Medicine at Mount Sinai, New York City, New York, United States; CUNY School of Public Health, Astoria, New York, United States; Icahn School of Medicine at Mount Sinai, New York City, New York, United States; Icahn School of Medicine at Mount Sinai, New York City, New York, United States; Johns Hopkins, University, Baltimore, Maryland, United States; Johns Hopkins University School of Medicine, Baltimore, Maryland, United States

## Abstract

The Centers for Medicare and Medicaid Services’ (CMS) Acute Hospital Care at Home waiver offers hospital-level reimbursement to provide acute hospital-level care in patients’ homes for the first time. While this initiative may make acute care at home more financially viable for health systems, it also requires aligning Hospital at Home (HaH) operations with inpatient, rather than outpatient, regulatory requirements. We aimed to understand how participating HaH programs adapted to these requirements. We conducted semi-structured interviews with multiple leaders from 14 HaH waiver programs (n=18 clinical/medical, operational and program directors) varying in size, urbanicity, structure, and region, examining data through thematic analysis. Both urban and rural participants described geographic effects of waiver requirements. For instance, to ensure response to patient emergencies within 30 minutes, programs contracted with paramedic services to expand service areas, added program locations or moved primary locations to other system hubs. Programs maximized staff capacity across service areas by “leasing” staff from other home-based programs, focusing on urban hubs with more staff, balancing in-person visits with remote monitoring, and providing “hybrid” in-person/video appointments. However, travel time, length of acute care visits, staffing shortages, the need for new skills (e.g., acute care nurses, dietitians) and limited state scope of practice regulations, particularly for paramedics, limited the area and populations served. Adapting to waiver requirements required significant efforts to address staffing, logistical and regulatory challenges. Future waiver improvements should explicitly consider the unique resources needed to expand hospital-level care in geographically diverse ambulatory environments.

